# Role of oncostatin-M in ECM remodeling and plaque vulnerability

**DOI:** 10.1007/s11010-023-04673-8

**Published:** 2023-03-01

**Authors:** Parth Patel, Vikrant Rai, Devendra K. Agrawal

**Affiliations:** Department of Translational Research, College of Osteopathic Medicine of the Pacific, Western University of Health Sciences, 309 E. Second Street, Pomona, CA 91766-1854 USA

**Keywords:** Atherosclerosis, Chronic inflammation, ECM remodeling, Oncostatin-M, Plaque vulnerability, Therapeutic target

## Abstract

Atherosclerosis is a multifactorial inflammatory disease characterized by the development of plaque formation leading to occlusion of the vessel and hypoxia of the tissue supplied by the vessel. Chronic inflammation and altered collagen expression render stable plaque to unstable and increase plaque vulnerability. Thinned and weakened fibrous cap results in plaque rupture and formation of thrombosis and emboli formation leading to acute ischemic events such as stroke and myocardial infarction. Inflammatory mediators including TREM-1, TLRs, MMPs, and immune cells play a critical role in plaque vulnerability. Among the other inflammatory mediators, oncostatin-M (OSM), a pro-inflammatory cytokine, play an important role in the development and progression of atherosclerosis, however, the role of OSM in plaque vulnerability and extracellular matrix remodeling (ECM) is not well understood and studied. Since ECM remodeling plays an important role in atherosclerosis and plaque vulnerability, a detailed investigation on the role of OSM in ECM remodeling and plaque vulnerability is critical. This is important because the role of OSM has been discussed in the context of proliferation of vascular smooth muscle cells and regulation of cytokine expression but the role of OSM is scarcely discussed in relation to ECM remodeling and plaque vulnerability. This review focuses on critically discussing the role of OSM in ECM remodeling and plaque vulnerability.

## Introduction

**Fig. 1:**
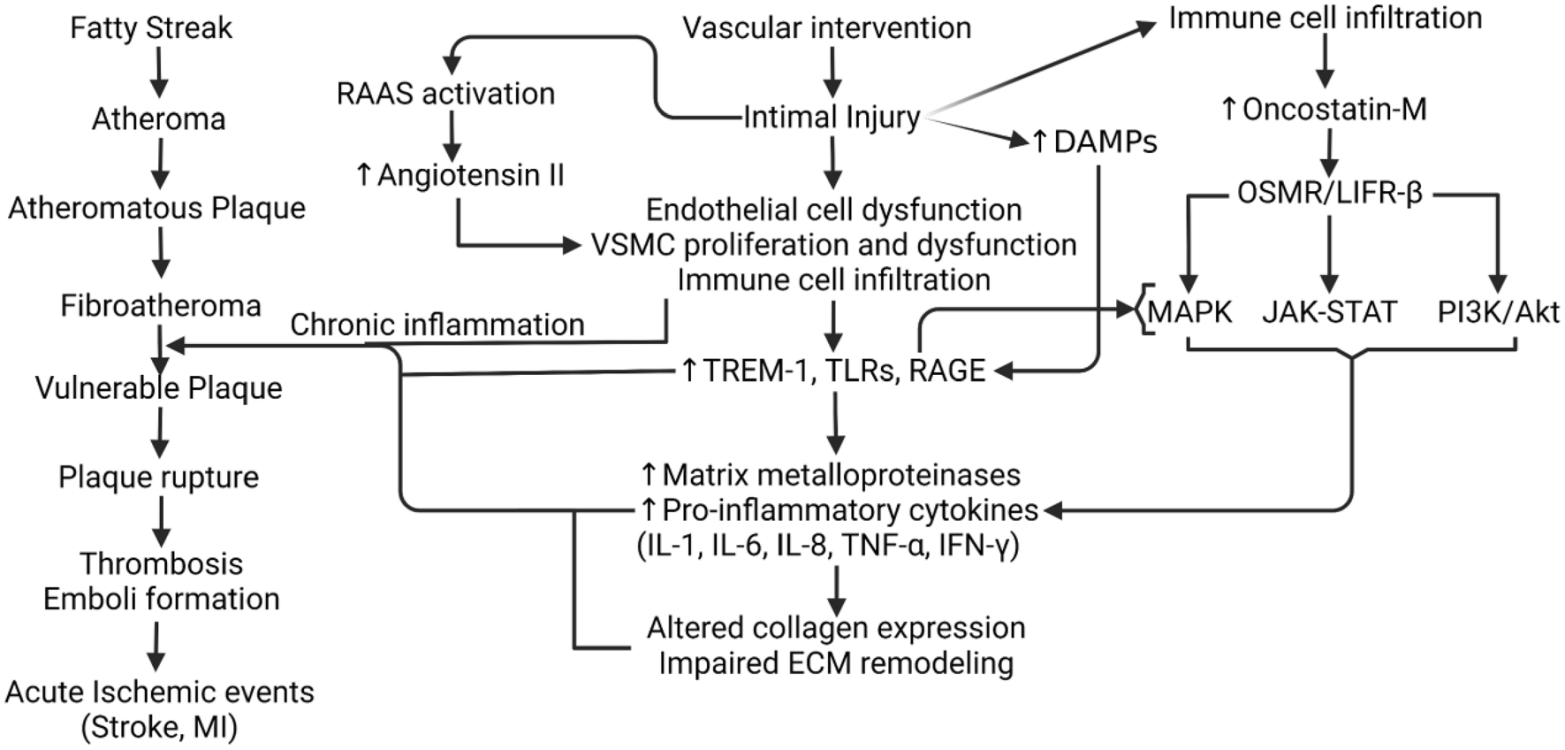
Cellular, molecular, and pathological events involved in the underlying pathogenesis of plaque formation, progression, and rupture. Renin angiotensin aldosterone system (RAAS), vascular smooth muscle cells (VSMCs), triggering receptor expressed on myeloid cells-1 (TREM-1), toll-like receptor (TLRs), receptor for advanced glycation end products (RAGE), interleukin (IL), interferon (IFN), extracellular matrix (ECM), damage-associated molecular patterns (DAMPs), oncostatin-M receptor (OSMR), leukemia inhibitory factor receptor (LIFR)-β, mitogen-activated protein kinase kinase (MEK), extracellular signal-regulated kinase (ERK), Janus kinase/signal transducers and activators of transcription (JAK/STAT), Mitogen-Activated Protein Kinase (MAPK)

Atherosclerosis is one of the leading causes of death across the globe [1]. This vascular inflammatory disease is caused by the buildup of plaque in vasculature throughout the body which, upon rupture can cause acute outcomes like stroke and myocardial infarction (MI) [2]. Plaque in simple terms is a deposition of lipids and scar tissue inside vasculature which may partially or wholly occlude the vessel. In addition to the fatty streak formation leading to plaque formation, plaque may also be formed in response to intimal injury after intravascular interventions (Fig. 1). Inflammation of the intima and media in response to endothelial damage promotes the modification of lipoproteins as well as recruit macrophages, the instigators of inflammation. The intimal injury also induces local activation of angiotensin II and renin–angiotensin–aldosterone system, and this may also lead to neointimal formation and hyperplasia. Chronic inflammation, activation of inflammatory mediators, alteration in collagens, endothelial dysfunction, and proliferation of vascular smooth muscle cells (VSMCs) all together mediate plaque progression and vulnerability [3–8] (Fig. 1). Vulnerable plaques are prone to rupture. Ruptured plaques may interact with thrombotic factors leading to thrombosis at the site of plaque rupture [9]. Depending on the size as well as the composition of the plaque, it can form either a thrombotic- or athero-emboli which can lead to peripheral vessel occlusion [10]. Plaque formation plays an essential role in neurological vessel occlusion as emboli can form at a different site in the body yet travel and ultimately occlude the much narrower vessels of the brain causing a stroke. Myocardial infarction is caused by occlusion of coronary vessels involving similar pathogenesis of plaque formation and rupture [11] (Fig. 1).

Plaque rupture is the precursor event for thrombosis and emboli; thus, it is important to understand the nature of plaque. Plaques are of two types: stable and unstable [12]. Unstable/vulnerable plaques are prone to rupture, and this is due to the different structural and cellular composition of stable and vulnerable plaques. Vulnerable plaques are primarily characterized by a lipid-rich necrotic core, a thin fibrous cap, and chronic infiltration of inflammatory immune cells [13]. The stability of the plaque is influenced by various factors such as inflammation and remodeling of extracellular matrix (ECM). Pro-inflammatory immune cells and cytokines make plaque vulnerable to rupture through the secretion of enzymes and metabolites that destabilize the plaque [3]. Additionally, increased expression of inflammatory surface receptors including triggering receptor expressed on myeloid cells (TREM)-1, toll-like receptor (TLR)-4, the receptor for the advanced glycation end product (RAGE), vitamin D deficiency, damage-associated molecular proteins (DAMPs), and S100 proteins also play a significant role in plaque progression and vulnerability [4, 5, 14–16] (Fig. 1). TREM-1, a receptor expressed by polymorphonuclear neutrophils (PMNs) and macrophages, are involved in the inflammatory response and plaque vulnerability as demonstrated by an increased expression in symptomatic human atherosclerotic plaques. TNF-α- regulated increased TREM1 expression mediates activation of matrix-metalloproteinases (MMP)-1 and MMP-9, which degrade collagens and reduce the structural integrity of the plaque, leading to increased plaque vulnerability [4–6]. Additionally, the formation of foam cells with increased recruitment of monocytes in the presence of lipid-rich lipoproteins such as low-density lipoproteins (LDL), increased oxidative stress, MMPs, ECM remodeling, and inflammatory cytokines enhance plaque vulnerability [17–19]. These studies suggest that plaque development, progression, and vulnerability are multifactorial but chronic inflammation is a common denominator.

Oncostatin-M (OSM) is an IL-6 family inflammatory cytokine and plays a critical role in the development and progression of the underlying pathogenesis of atherosclerosis but the role of OSM in the context of plaque vulnerability and ECM remodeling has not been discussed in detail in the literature (Fig. 1). [20–25]. OSM signals through leukemia inhibitory factor receptor (LIFR)-β or oncostatin-M receptor (OSMR)-β, a receptor coupled to a gp130 subunit. This further selectively activates the mitogen-activated protein kinase kinase (MEK)-extracellular signal-regulated kinase (ERK) and Janus kinase/signal transducers and activators of transcription (JAK/STAT) pathways [20, 26]. OSM, like the other cytokines in the IL-6 family, can increase phosphorylated STAT3 dimers and promote transcription of a variety of genes involved in activities ranging from induction of inflammation to endothelial cell injury [27] (Fig. 1). Given that OSM exhibits many of its pro-inflammatory and atherosclerotic effects through the JAK/STAT pathway, modulating activity of JAK/STAT pathway may be a viable method of attenuating the atherosclerotic effects of OSM [27]. However, the underlying mechanism of OSM-mediated plaque vulnerability and its role in ECM remodeling after an intimal injury is largely unknown. As a response to plaque buildup and myointimal injury due to the inflammatory response, the blood vessel can undergo “inward remodeling” leading to a decrease in luminal size. Vascular remodeling involves ECM remodeling involving MMPs and this suggests that plaque buildup involving MMPs itself may affect ECM remodeling process. [28].

Matrix metalloproteinases (MMPs), a group of enzymes that encompass both proteases and elastases, are involved in the inflammatory response and degrade ECM components [29, 30]. MMPs can also have direct effects on plaque stability via degradation of the fibrous cap and make it prone to rupture [31]. After plaque rupture, the lipid-rich core of the plaque is exposed to the blood allowing interaction with thrombotic factors and subsequently, platelet aggregation [9] resulting in thrombus formation. MMPs by their direct or indirect effects regulate plaque stability and via degradation of ECM elements such as collagen and elastin promote plaque destabilization [28, 31]. OSM which upregulates MMP-9 through Mitogen-Activated Protein Kinase Kinase-Extracellular Signal-Regulated Kinase Pathway (MAPK-ERK) may be a key molecule in plaque destabilization as it promotes foam cell formation, a cell type that can secrete MMPs, and activate MMPs [25, 32] (Fig. 1). This suggests an important role of OSM in plaque formation, progression, and vulnerability; however, how OSM regulates ECM remodeling involving elastases and proteases is scarcely discussed. Further, the effect of inhibiting OSM and its effect on plaque vulnerability has not been studied. This review focuses on critically discussing the regulation of OSM-mediated ECM remodeling and the molecular and cellular factors involved in this process.

## OSM, inflammation, and plaque stability

**Fig. 2:**
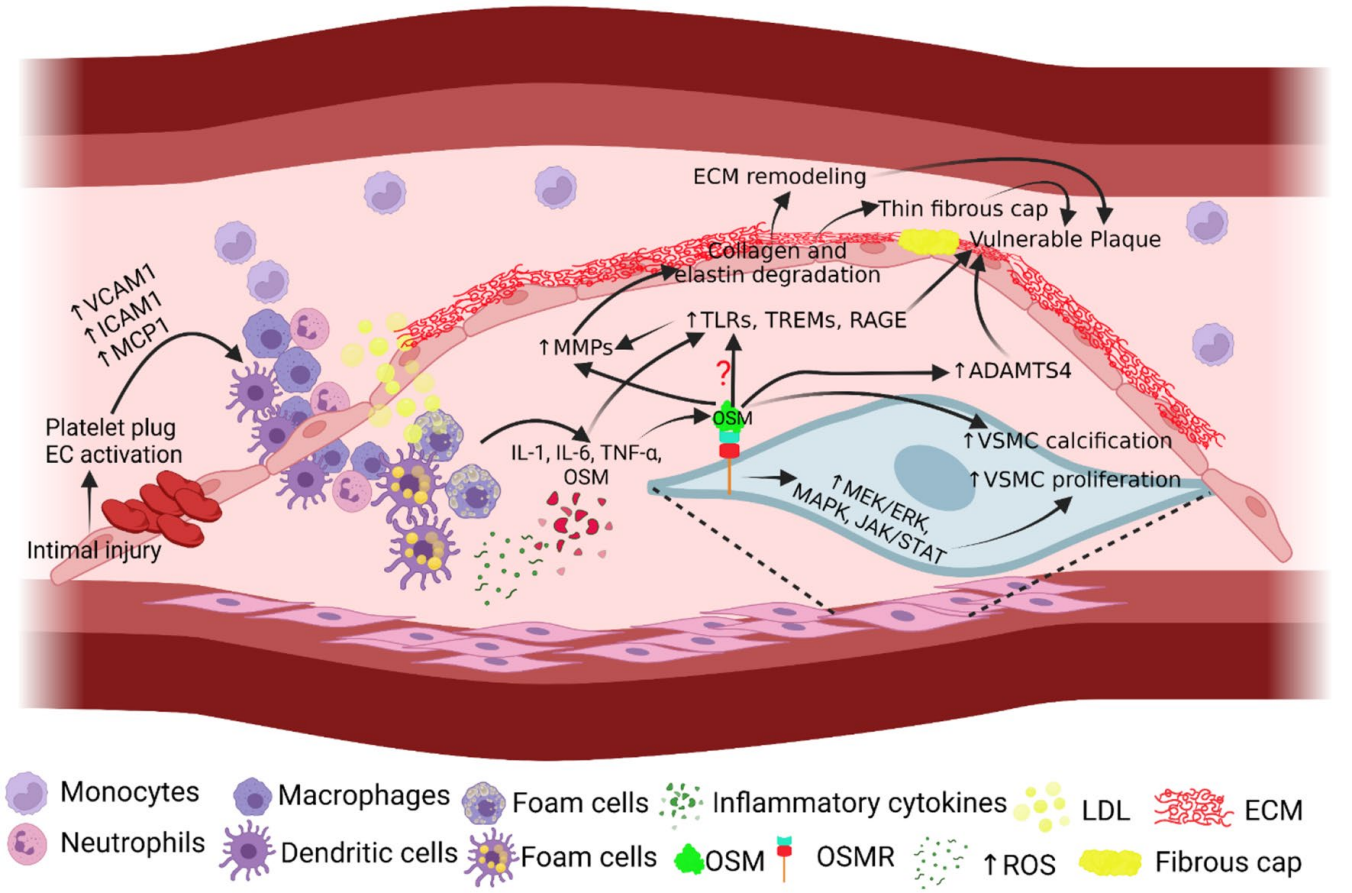
Schematic diagram showing potential mechanisms underlying Oncostatin-M (OSM)-mediated plaque vulnerability. Activation of endothelial cells (ECs) and platelet plug formation after injury increase secretion of adhesion molecules and chemokines resulting in recruitment of immune cells. Infiltrated immune cells secrete pro-inflammatory cytokines including OSM and increase expression of matrix-metalloproteinases (MMPs). OSM also activates cytoplasmic signaling leading to proliferation and calcification of vascular smooth muscle cells (VSMCs). OSM also activates ADAMTS4. These mechanisms may contribute to extracellular matrix (ECM) remodeling and thinning of the fibrous cap resulting in plaque vulnerability. Activation of surface receptors like triggering receptor expressed on myeloid cells (TREMs), toll-like receptors (TLRs), and receptor for advanced glycation end products (RAGE) may also contribute to plaque vulnerability but how OSM activate these receptor warrants investigation. Monocyte chemoattractant protein-1 (MCP-1), intercellular adhesion molecule-1 (ICAM-1), vascular cell adhesion molecule-1 (VCAM-1), low-density lipoproteins (LDL), reactive oxygen species (ROS), ADAMTS4 (a disintegrin and metalloproteinase with thrombospondin motifs 4), mitogen-activated protein kinase kinase (MEK)- extracellular signal-regulated kinase (ERK), Janus kinase/signal transducers and activators of transcription (JAK/STAT), oncostatin-M receptor (OSMR), interleukin (IL), and tumor necrosis factor (TNF)-α

Atherosclerosis has been described as both a cardiovascular and inflammatory disease due to the involvement of inflammatory mediators in plaque formation, calcification, and vulnerability [33]. Along with abnormal lipid accumulation and turbulent blood flow, endothelial dysfunction and activation of endothelial cells (ECs) also play a critical role in plaque formation [34]. OSM induces ECs in APOE*3Leiden.CETP mice vasculature [35]. Furthermore, ECs have the greatest expression of OSM receptors amongst all other normal tissues and OSM stimulates the reprogramming of ECs [36]. This points to OSM likely being one of the initiating molecules in atherosclerosis development. The factors secreted by activated ECs lead to the attraction and adhesion of inflammatory cells. Recruitment and adhesion of inflammatory cells, mainly macrophages, is mediated by oxidized LDL (oxLDL) and increased expression of monocyte chemoattractant protein-1 (MCP-1), intercellular adhesion molecule-1 (ICAM-1), and vascular cell adhesion molecule-1 (VCAM-1) which play an important role in the development of atherosclerosis (Fig. 2). Recruited immune cells, mainly monocytes differentiated to macrophages, upon uptake of ox-LDL, become foam cells and contribute to plaque development and progression [33, 34, 37, 38]. TNF-α, playing a critical role in plaque vulnerability, has been shown to synergistically increase IL-6 and MCP-1 production in conjunction with OSM, both key modulators of inflammation and subsequent plaque formation [5, 39] (Fig. 2). Further, the concept that OSM mediates plaque vulnerability is supported by the fact that OSM and TNF-α colocalize in atherosclerotic plaques and TNF- α plays a critical role in early as well as late stages of atherosclerosis [40–42]. This indicates another possible pathway by which OSM exhibits atherogenic effects. MCP-1 in APOE*3Leiden.CETP mice ECs have also been shown to become elevated directly in response to OSM treatment [35]. Additionally, OSM treatment in the vasculature of these mice resulted in elevated ICAM mRNA as well [35].

Given the effect of OSM in modulating molecules such as MCP-1 and ICAM that play a role in immune cell recruitment, OSM may play a significant role in regulating the pro-inflammatory processes that preclude plaque formation. OSM is also a secreted product of macrophages infiltrating intimal injury sites as well as VSMCs surrounding blood vessels that may also have a role in plaque formation [22]. OSM has been associated with the neovascularization of plaque, specifically when expressed in SMCs, providing further evidence of supporting plaque formation [23]. The role of OSM in plaque vulnerability is further supported by the findings that variant rs1316887 in the OSMR locus is associated with increased plaque vulnerability [43]. OSM may also play a role in plaque vulnerability by increasing the expression of ADAMTS4 (a disintegrin and metalloproteinase with thrombospondin motifs 4) [44], a marker for plaque vulnerability. Another modality to enhance plaque stability may be by increasing plaque calcification because OSM promotes osteoblast differentiation in bone [45]. However, investigating the type of calcification induced by OSM is worthy because macrocalcification induces plaque stability while microcalcification induces plaque rupture. This notion is further supported by the fact that OSM induces VSMC calcification and VSMC plasticity and heterogeneity is an attractive target in thrombosis, stenosis, and plaque stability. Although VSMCs differentiation is beneficial for plaque stability but aberrant proliferation of VSMCs, VSMC-derived macrophage-like cells, and differentiated VSMCs lacking VSMC markers may contribute to plaque vulnerability. OSM promotes osteoblastic differentiation of human VSMCs through JAK3-STAT3 pathway [46–49]. This is supported by the fact that inhibition of the JAK2/STAT3 activation in macrophages in mice with OSMR-β deficiency is protective in atherosclerosis [20] (Fig. 2). All these studies suggest a possible role of OSM in inducing plaque vulnerability but there is no available literature in this context delineating a direct causal-effect relationship or documenting that OSM inhibition attenuates plaque vulnerability.

Cytokines secreted by inflammatory cells can mediate chemotaxis, differentiation, and cell activation allowing the amplification of even a minor inflammatory event. They can also lead to plaque vulnerability by modulating cellular processes such as ECM remodeling as well as changing plaque composition via modification of plaque components. The key components of atherosclerotic plaque include a necrotic core composed of dead cells as well as a fibrous cap composed of collagen and other ECM components [50]. OSM/OSMR is expressed on VSMCs and upregulates MMP-9. MMP-9 is an ECM remodeling enzyme that can degrade the fibrous cap of atherosclerotic plaque through the ERK pathway and makes plaque unstable [25, 31] (Fig. 2). This suggests a probable role of OSM in plaque vulnerability mediated by MMP-9 by collagen degradation and weakening of the structural integrity of the plaque. In addition, OSMR-β knockout mice exhibited increased plaque stability measured by multiple metrics including elevated collagen and a significantly smaller necrotic core [20]. This further implicates OSM as a key regulatory component modulating plaque structure and supports the notion that inhibition of OSM signaling may have therapeutic potential. These studies suggest a direct role of OSM in modulating plaque instability and indicate that OSM is likely an upstream regulator of plaque formation, progression, and vulnerability.

## OSM and ECM remodeling

The ECM of blood vessels can be thought of as the superstructure on which the vascular layers, the adventitia, media, and intima, are built. It consists of numerous components however collagen and elastin make up the bulk of the matrix. A specific aspect of the ECM that is of particular importance is its ability to change, both due to general aging and also in response to vascular pathologies such as atherosclerosis [51]. Enzymes essential for dynamic characteristics of ECM include MMPs, elastases, and proteases which can modify and digest the structural molecules and constituents of ECM [51]. Of note however is that the ECM is not the only producer of enzymes related to ECM remodeling, but the macrophages recruited after increased MCP-1 expression after EC activation [37, 52]. Foam cells in the plaque can also secrete MMPs [18]. Another cell type, neutrophils, can generally release collagenases, MMPs, as well as neutrophil elastases upon activation at the site of inflammation [51, 53] (Fig. 2). Persistent inflammation and immune cell recruitment mediate ECM remodeling which is an essential part of vascular remodeling. Additionally, given the fact that the vascular ECM is not the only source of proteolytic enzymes, it can be surmised that the effects of these enzymes, whether for extravascular sources or intravascular sources, may exhibit effects on the resulting atherosclerotic plaque as well.

The necrotic core and the fibrous cap are two of the key components comprising atherosclerotic plaque. To see how ECM remodeling affects the stability of the plaque, it is necessary to see how ECM remodeling modifies the structural components of plaque. Collagen, an important load-bearing component of the ECM is one of many cellular elements whose degradation decreases the stability of the plaque [54]. MMPs released by both VSMCs, as well as macrophages in response to ECM remodeling, can degrade the fibrous cap thereby increasing plaque vulnerability. MMP1 and MMP-13 released by macrophages as well as MMP 2 released by VSMCs are primarily collagenases that directly degrade collagen in the fibrous cap [52, 54–56] (Fig. 1). Further, MMP-1 and MMP-9 are involved in plaque destabilization via p38-MAPK (activated by OSM; Fig. 1) and JNK signaling and MMPs, whose secretion is regulated by OSM, regulates ECM remodeling [57]. These interactions suggest a critical role of OSM in ECM remodeling that warrants future research.

Elastin is another key ECM component playing a critical role in plaque vulnerability and ECM remodeling [28]. Since plaque rupture results in symptomatic atherosclerosis, patients with preoperative symptoms are more likely to be characterized as having unstable plaques [58]. The results of the study by Asciutto et al. [59] comparing elastin levels in preoperative symptoms and asymptomatic patients showed that lower elastin levels in plaques were associated with preoperative symptoms thereby supporting the notion that elastin is likely an important factor in determining plaque stability [59]. OSM is involved in ECM remodeling through lysyl oxidase like-2 which regulates collagen and elastin [60]. Moreover, elastase secreted from infiltrating neutrophils as well as MMP-2 secreted from VSMCs exhibit elastin degrading abilities [52, 61]. In particular, high levels of neutrophil elastase were found in infiltrating shoulders of atherosclerotic plaque, an area that is highly susceptible to rupture and increases the likelihood of acute coronary syndromes [61]. These results provide evidence of a possible mechanism of action for elastases to increase the likelihood of plaque rupture by the metabolism of the ECM component elastin in rupture-prone regions of the plaque. This process can be perpetuated by the pro-inflammatory cytokines secreted by OSM/OSMR activation and thus targeting OSM-OSMR signaling might be therapeutic.

Upstream regulation of ECM remodeling occurs through a variety of pathways, for example, through Angiotensin II. It has already been established that Angiotensin II is released in response to intimal injury. Angiotensin II has been shown to impact ECM remodeling through the Ang II-Ets1-CREG axis [62]. In VSMC, Angiotensin II reduces the expression of CREG, a cellular repressor of genes involved in vascular remodeling, through Ets-1 leading to increased medial area and collagen deposition [62]. Additionally, Ets-1 has also been directly related to the regulation of ECM remodeling enzymes with the discovery of an Ets-1 binding site on MMP promoter regions only further adding to the possible pathways through which Angiotensin II affects vascular remodeling [7]. Interestingly, activation of ACE via OSM on vascular walls [63] provides a possible evidence through which OSM modulates ECM remodeling, and thus, plaque vulnerability. The notion that OSM may regulate ECM remodeling is supported by the fact that OSM regulates fibroblast function via STAT-3 [64] and fibroblasts acquiring myofibroblast phenotype secretes collagens and play an important role in ECM remodeling [65, 66].

## Potential therapeutic targets

Having discussed numerous proteases, cytokines, and cellular receptors implicated in plaque progression and vulnerability, the next step is to evaluate their possible therapeutic potential. Immune cells including macrophages, dendritic cells, lymphocytes, and neutrophils infiltrated after intimal injury and plaque development contribute to various vascular pathologies including plaque vulnerability [4, 67–70]. T-cell immunoglobulin and mucin domain (TIM) proteins are expressed in a variety of T-cells, a cell type shown to module neointimal hyperplasia, thrombosis, stenosis, plaque formation, and atherosclerosis [67, 68, 71]. Of note is TIM-3, a specific marker expressed on macrophages and neutrophils, two cell types that have been discussed to play a very important role in the initiation and progression of atherosclerosis. Increased lesion size and macrophage infiltration in mice with anti-TIM3 Ab indicate a possible protective effect of TIM in the progression of atherosclerosis [71], although plaque stability was not measured.

Another molecule expressed by macrophages and neutrophils, TREM-1, is known as an upstream regulator of cell activation and inflammation and plays a critical role in the pathogenesis of inflammation [72, 73]. Again, the strong association of atherosclerosis with inflammation warrants a deeper look into the multitude of inflammatory pathways to find effective therapeutic targets. Joffre et al. [74] investigated the effect of TREM-1 inhibition on plaque development via both genetically engineered mice with double knockout of TREM-1 as well as pharmacological inhibition of TREM-1 using LR-12 peptide. Both methodologies resulted in a reduction in size and level of inflammation in atherosclerotic plaque [74]. This establishes TREM-1 as not only a regulator of inflammation but also potentially as a modulator of atherosclerosis via macrophage and neutrophil activation. However, investigation and characterization of the pathways and targeting TREM-1 to exert its protective effects in atherosclerosis must be done to solidify its potential as a target of clinical significance. Targeting TREM-1 to attenuate plaque vulnerability is the interest of the research community [72, 74–76], however, no literature suggests any relationship between OSM and TREM-1. The basis of this notion is that TNF-α regulates TREM-1 [5] and OSM regulates TNF-α-mediated inflammation [77]. However, how OSM and TREM-1 regulate each other warrants further investigation (Fig. 1).

MMPs play an important role in the initiation and progression of atherosclerosis through varied pathways including secretion from macrophages attracted to the lesion site as well as through activation of the ECM remodeling pathway in response to vascular injury. Data from a prospective study shows a strong association between MMP levels, specifically MMP-9, with an increase in total plaque volume [78]. This establishes a connection between MMP levels and plaque progression. It is then necessary to look at whether direct modulation of MMPs has any effect on lesion progression. Results from a study on ApoE and MMP-10 deficient mice showed a significant reduction in atheroma size for the MMP10 deficient group as well as a decrease in plaque macrophage content [79]. This shows that targeting MMPs may be a viable method to reduce either atherosclerosis progression or decrease the likelihood of symptomatic atherosclerosis [80, 81]. However, although these studies show the promise of MMPs as a therapeutic target, the differential effects of different MMPs must be considered when determining which MMPs to target. Further, as discussed above, OSM regulates MMPs, it will be worth investigating if inhibiting OSM will modulate MMPs and attenuate plaque vulnerability.

IL-1β is a pro-inflammatory cytokine secreted by macrophages and endothelial cells that can act to alter endothelial cell receptor morphology to attract additional monocytes [82]. A study by Bhaskar et al. utilized XMA 052, an anti-IL-1β antibody, in ApoE deficient mice to investigate its effect on plaque progression. Reduced plaque formation and macrophage infiltration in the lesions [82] provide evidence for IL-1β as a potential therapeutic target. This opens the possibility for other regulatory molecules that have similar effects in vitro to be considered when determining alternative therapeutic targets.

Conclusively, the above studies suggest that along with the mediators of inflammation involved in plaque formation and vulnerability and ECM remodeling, OSM might be therapeutic as well as a diagnostic target in cardiovascular diseases including atherosclerosis. However, the focus should be on establishing the role and signaling pathways of OSM mediating plaque vulnerability. The notion of using OSM as a diagnostic biomarker should also be considered as OSM has been reported as a biomarker in cardiovascular and other inflammatory diseases [22, 83–85].

## Conclusion

Atherosclerosis has been established as a chronic inflammatory disease with numerous cellular pathways involved in the initiation, progression, and vulnerability. Important cell types and molecules including cellular cytokines such as IL-6, TNF-α, IL- β, macrophages, neutrophils, and MMPs regulate ECM remodeling. Cellular processes such as ECM remodeling in response to a vascular injury activate a cascade of events that not only leads to the secretion of proteolytic proteins from the vasculature but also leads to the attraction and activation of cells from the immune system which also secrete ECM remodeling related enzymes. These two systems combine to exert destabilizing effects on plaque via the degradation of ECM components that are correlated with increased stability. However, the pathways through which their effects are exhibited are still not fully understood. This means that there are still potentially underexplored molecular targets such as OSM that may play a role in atherosclerotic pathogenesis, ECM remodeling, and plaque vulnerability. OSM plays a role in atherosclerosis and its protective and detrimental effect is context-dependent, more research is warranted to investigate whether clinically targeting OSM and its downstream signaling directly has any effect on plaque vulnerability and ECM remodeling. The multifactorial effects of these molecules make it difficult to irrefutably prove that they have a specific effect on plaque morphology which is one of many reasons that there are limited reports providing definitive evidence of OSM on the protective or detrimental effects on the progression of atherosclerosis, plaque vulnerability, and ECM remodeling.

## Data Availability

Not applicable since the information is gathered from published articles.
